# Peripheral Pulmonary Torsion in the Emphysematous Lingular Segment: A Case Report

**DOI:** 10.70352/scrj.cr.25-0495

**Published:** 2025-10-29

**Authors:** Yusuke Kita, Kazuki Hayashi, Jun Hanaoka

**Affiliations:** 1 Department of General Thoracic Surgery, Omi Medical Center, Kusatsu, Shiga, Japan; 2 Department of Thoracic Surgery, Shiga University of Medical Science, Otsu, Shiga, Japan

**Keywords:** lung torsion, peripheral, emphysematous change

## Abstract

**INTRODUCTION:**

Partial pulmonary torsion is extremely rare.

**CASE PRESENTATION:**

A 29-year-old man was initially treated with antibiotics for presumed bacterial pneumonia. Despite treatment, his inflammatory condition did not improve, and increasing pleural effusion was suggestive of empyema, leading to referral to our department. Following drainage, the patient’s condition did not improve. CT showed an increase in pleural effusion, with infiltrative shadows remaining within a clearly demarcated area, along with emphysematous changes in both lungs. He was diagnosed with lung abscess and empyema, and surgery was performed. Intraoperatively, the peripheral lingular segment appeared dark reddish, firm, and hyperlobulated. The affected area could be bluntly dissected, with its central portion narrowed but continuous with the central lingular segment, which rotated at least 360°. Peripheral pulmonary torsion was diagnosed, and a partial resection was performed. Postoperative recovery was uneventful.

**CONCLUSIONS:**

Given the extreme rarity of peripheral pulmonary torsion, this case highlights the importance of considering torsion in persistent localized infiltrates unresponsive to treatment.

## INTRODUCTION

Pulmonary torsion is categorized as iatrogenic, traumatic, and spontaneous. The most common form is torsion of the remaining middle lobe following right upper lobectomy, with an incidence of approximately 0.1%.^1,2)^ Spontaneous torsion can be caused by pleural effusion, atelectasis, pneumothorax, and tumors.^3)^ Pulmonary torsion typically involves a lobe or the whole lung, and peripheral pulmonary torsion, occurring distal to the segmental bronchi, is extremely rare. We report a very rare case in which peripheral pulmonary torsion in the lingular segment was revealed during surgery.

## CASE PRESENTATION

A 29-year-old Finnish man with no previous medical history presented with fever and left-sided chest pain. There was no medical history, family history, or smoking history. A chest radiograph revealed infiltration in the left lower lung field, and he was initially treated with antibiotics for presumed bacterial pneumonia. However, his inflammatory condition did not improve, and increasing pleural effusion raised concern for empyema, prompting referral to our department.

**Fig. 1:**
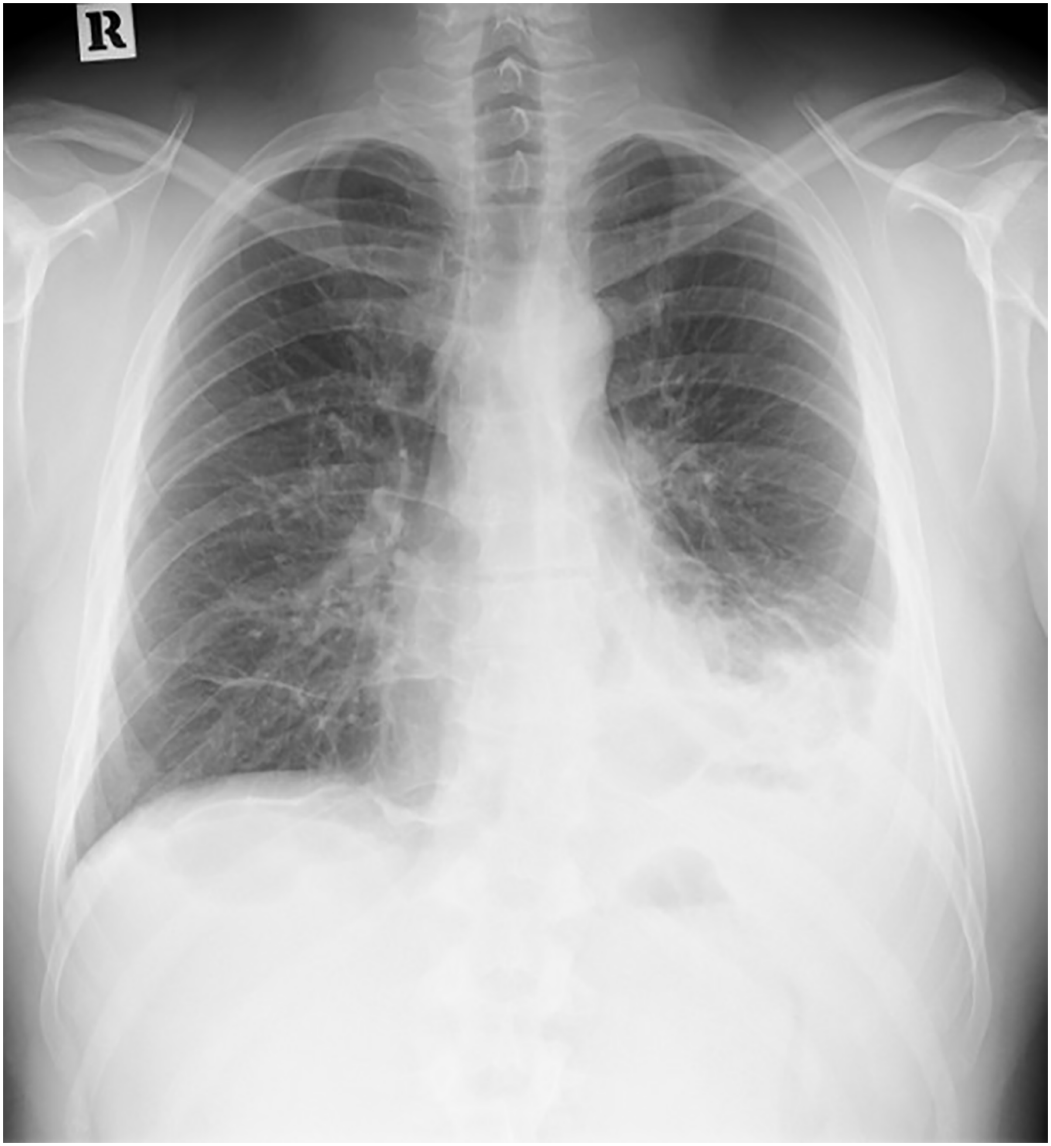
Chest radiograph revealed a pulmonary infiltrate in the left lower lung field and increased pleural effusion.

**Fig. 2:**
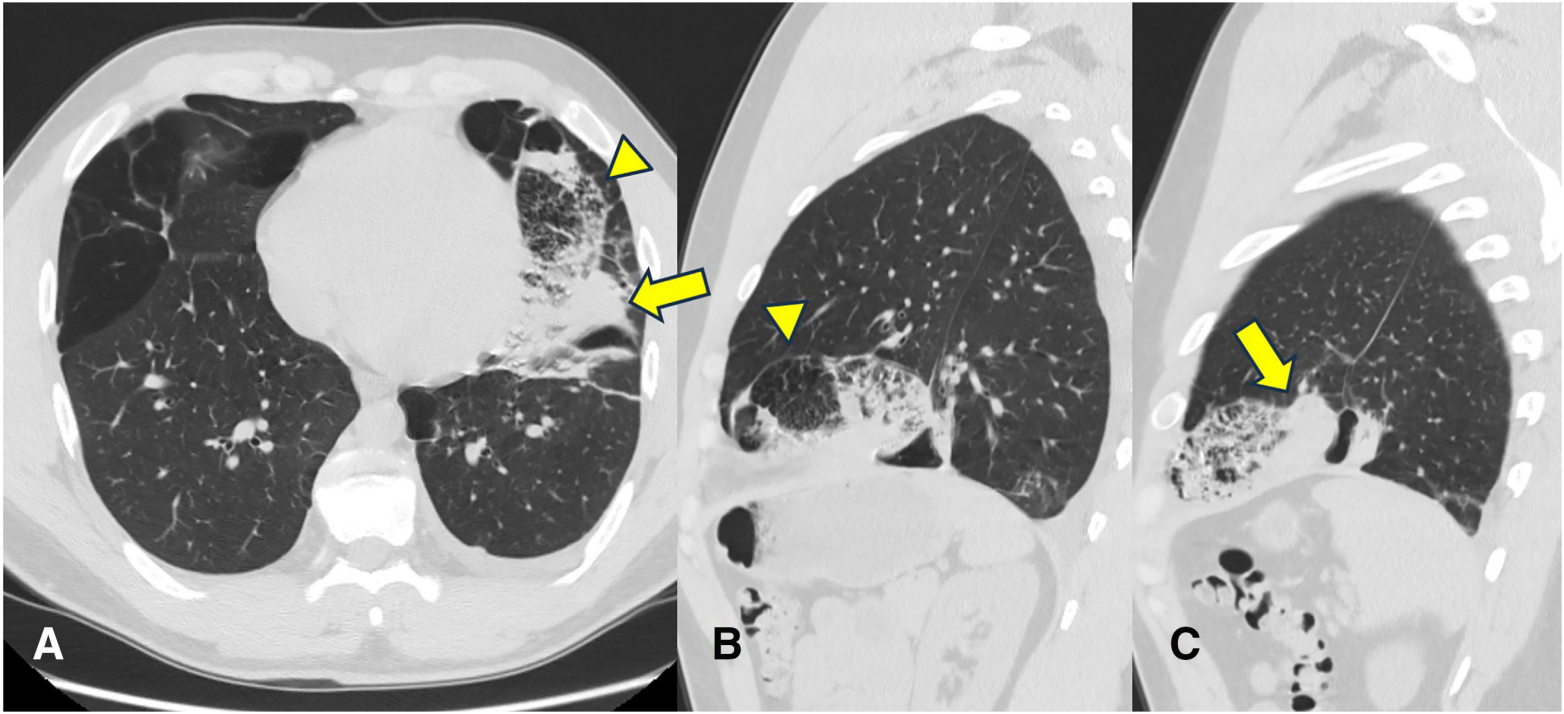
CT (**A**: axial, **B**, **C**: sagittal) images demonstrated a localized infiltrative lesion on the distal side of the lingular S5 segment, featuring lines indicative of hyperlobulation (arrow head) and a convex area of increased density outside this area (arrow).

A chest radiograph showed infiltration in the left lower lung field and increased pleural effusion (**Fig. 1**). Additionally, CT revealed an infiltrative lesion in the distal side of the lingular S5 segment, with hyperlobulation, along with emphysematous changes in both lungs (**Fig. 2**). Laboratory tests revealed a high white blood cell count (WBC) of 10.6 × 10^3^/μL), C-reactive protein (CRP) of 11.44 mg/dL, and D-dimer of 2.2 μg/mL.

Thoracic drainage was performed, draining 900 mL of serous pleural fluid. The pleural fluid had a pH of 8.0 and glucose level of 95 mg/dL, so empyema was considered unlikely.^4)^ However, bacterial pleuritis could not be ruled out; thus, antibiotic treatment (Garenoxacin, Tazobactam/Piperacillin, Meropenem) was performed for a total of 14 days, inflammation was still severe, indicated by a WBC of 11.2 × 10^3^/μL and CRP of 24.04 mg/dL. Contrast enhanced CT findings showed that the infiltrate remained unchanged and localized, indicating obstructive pneumonia or a lung abscess. No twisted vessels as lung torsion or abnormal blood vessels as pulmonary sequestration were observed, and no thrombi were found in the lower limbs and the pelvis. As pleural effusion had increased, surgery was decided, considering the development of lung abscess and empyema. The pneumonia was localized to the lingular segment; thus, lingular segmentectomy and dissection of adhesions of the empyema were planned.

**Fig. 3:**
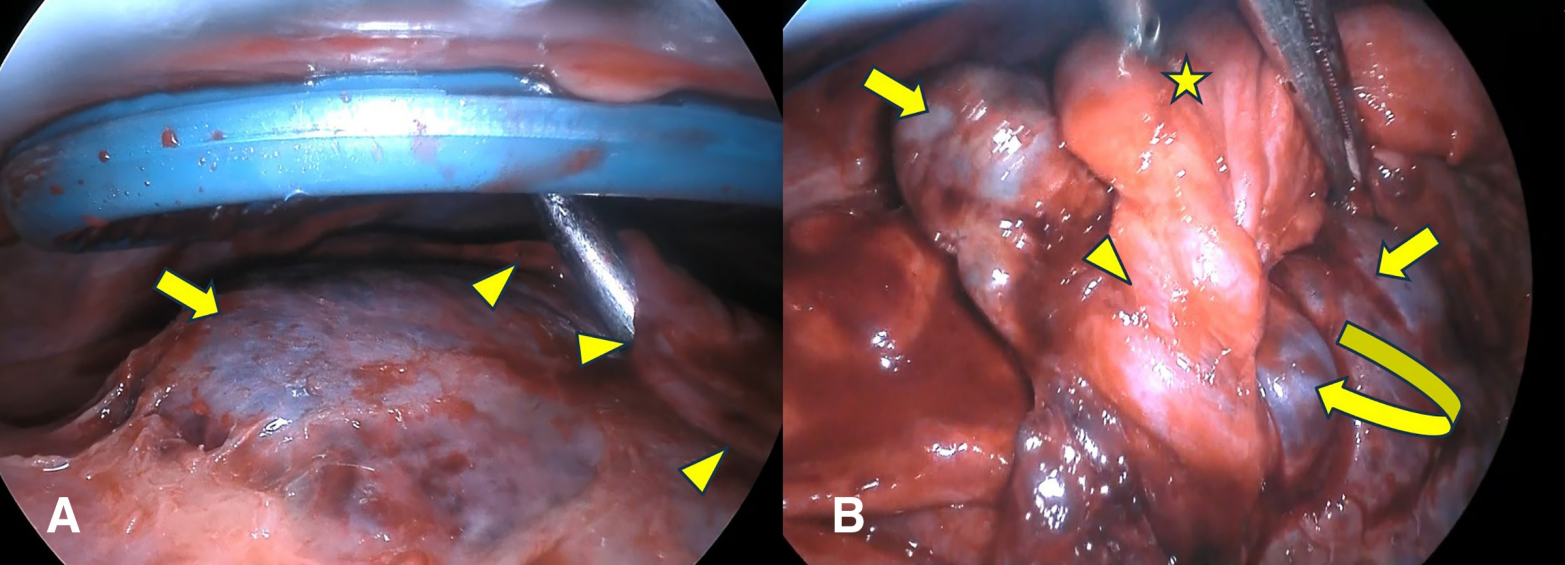
Intraoperatively, (**A**) the distal side of the lingular segment appeared dark reddish, firm (arrow). The area that appeared to be hyperlobulated on CT could be bluntly separated (arrow head). (**B**) The central portion narrowed but continued with the central lingular segment, which rotated at least to 360°. Normal area in the central lingular segment (star), the torsion site (arrow head), the distal side of the lingular segment (arrow).

Preoperative bronchoscopy did not reveal bronchial obstruction in the visible range up to the subsegmental bronchi. Further distal evaluation could not be performed. Video-assisted thoracic surgery was performed, revealing adhesions and purulent pleura mainly located on the diaphragm and dorsal side, which were dissected. The distal aspect of the lingular segment appeared dark reddish, firm, and hyperlobulated (**Fig. 3A**). This affected area could be bluntly separated, with its central portion narrowed but continued with the central lingular segment, which rotated at least 360° (**Fig. 3B**, **Movie**). Congestion and necrosis were observed distal to the torsion site, and no bronchial or vascular structures were observed on the lung surface. The main site of torsion was peripheral, not hyperlobulated. Based on these findings, peripheral pulmonary torsion was diagnosed. As detorsion was considered risky, partial resection using a stapler was performed in normal lung areas. The surgical time was 2 hours 39 min, and the blood loss volume was 30 mL. Postoperative recovery was uneventful. The chest drain was removed on POD 4, and he was discharged on day 13. No significant organisms were detected from tissue or pleural fluid cultures, and empyema was not identified. After discharge, anticardiolipin antibodies, lupus anticoagulant, protein C and S activity, and alpha-1 antitrypsin were measured waiting for inflammatory findings to improve, but no abnormalities were found.

**Fig. 4:**
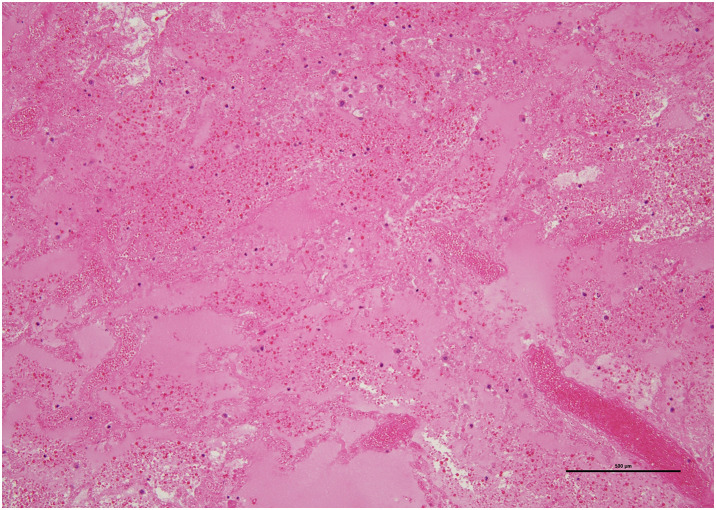
Microscopic examination showed evidence of hemorrhage, thrombus, and necrosis, accompanied by congestion, without signs of neutrophil infiltration and abscess formation.

Histopathological examination showed signs of hemorrhage, thrombus, and necrosis, accompanied by congestion (**Fig. 4**). No neutrophil infiltration or abscess formation was found, and pulmonary torsion led to the diagnosis of pulmonary infarction. Furthermore, bronchi and vascular structures were observed near the resection margin, which was continuous. In the peripheral, thinning and destruction of the alveolar walls were observed, indicating severe degeneration. So, no specific pathological examination was performed for emphysematous changes.

## DISCUSSION

Most pulmonary torsions involved one lobe or the whole lung. Although segmental pulmonary torsion has occasionally been reported,^5,6)^ peripheral pulmonary torsion is exceedingly rare, with only one similar case found in the literature.^7)^

Diagnosis is most definitive with contrast-enhanced CT,^6,8)^ which can detect twisted vessels and the bronchi. However, in the presented case, torsion occurred peripherally, and typical signs were absent, making preoperative diagnosis difficult. Although Shimizu et al.^7)^ were not mentioned, similar characteristic CT findings—including a peripherally bordered infiltrative shadow with an outwardly localized area of increased density—were observed in this case and may suggest peripheral pulmonary torsion. Compared with intraoperative findings, we considered that localized increase in density was the torsion point, the infiltrate shadow was a pulmonary infarction, and hyperlobulated was a lung overlap. Although inflow vessels into the torsion point were identified, the twisted vessels were not clearly seen, possibly because the lung had already collapsed. Furthermore, localized increase in density was observed mainly on the dorsal side, which was considered a gravitational change in pneumonia images; these findings led to no consideration of pulmonary torsion.

Possible causes include an incomplete interlobar or intersegmental fissure (such as an accessory posterior interlobar fissure)^6,7)^; however, these were not observed in this case. First, the cause of the torsion was presumed to be related to the anatomical characteristics of the peripheral lingular segment. This segment is not fixed, allowing easy movement, and gradually tapers toward the periphery. In addition, emphysematous changes were observed at the distal portion of this thinned area, and the expansion of the peripheral region had stretched the lung parenchyma, suggesting an anatomical predisposition to torsion. Second, emphysematous changes may have caused an unrecognized pneumothorax, and increased mobility and adhesions may have led to torsion. Third, pneumonia-induced atelectasis and pleural effusion may increase segmental mobility, predisposing to torsion. Furthermore, the increased weight of the affected area also can cause torsion according to the same principle as a tumor. However, the exact trigger for the initial torsion remains unclear.

Although thrombosis was found in the resected lung, torsion secondary to pulmonary infarction was considered unlikely. No thrombi were found on contrast-enhanced CT, and laboratory tests were negative, and no medical history, making a thrombus-related cause improbable. Therefore, the thrombi were determined to be secondary to the torsion.

Regarding the emphysematous changes observed on imaging, congenital lobar emphysema, congenital pulmonary airway malformation (CPAM), and Swyer–James–MacLeod syndrome could not be completely excluded; however, these conditions typically present unilaterally or involve a single lobe, and it would be unlikely for the patient to have remained asymptomatic until this age. In addition, the patient tested negative for alpha-1 antitrypsin deficiency and had no relevant past medical, family, or smoking history. Histologically, no ciliated columnar epithelium was identified, and the cyst morphology lacked features suggestive of CPAM. As far as could be confirmed, the pathological findings were consistent with nonspecific emphysematous changes. Therefore, the etiology of the emphysema remained undetermined.

Pulmonary torsion carries a high mortality risk because of thrombosis, necrotic debris, and infection.^3,9,10)^ Thus, prompt intervention is essential, with the main treatment being detorsion or resection. In this case, necrosis caused by torsion led to resection without detorsion, resulting in a favorable outcome.

## CONCLUSIONS

This study presents an extremely rare case of peripheral pulmonary torsion in the emphysematous lingular segment. This case underscores that when a localized infiltrate is observed within a clearly defined intersegmental region and does not respond to appropriate treatment, peripheral pulmonary torsion should be considered a possible cause. In such cases, early surgical intervention should be considered not only for definitive treatment but also for accurate diagnosis.

## SUPPLEMENTARY MATERIAL

Movie The distal aspect of the lingular segment appeared dark reddish; this affected area could be bluntly separated, with its central portion narrowed but continued with the central lingular segment, which rotated at least to 360°. The area distal to the torsion site was determined for congestion and necrosis. As detorsion was considered risky, partial resection using a stapler was performed at the forceps position.

## DECLARATIONS

### Availability of data and materials

The data supporting the conclusions of this article are included within the article.
